# Editorial: Insights in RNA: 2025

**DOI:** 10.3389/fgene.2026.1947303

**Published:** 2026-08-06

**Authors:** Hassan Dastsooz, Naoyuki Kataoka, Nicoletta Potenza

**Affiliations:** 1 Department of Life Sciences and Systems Biology, The University of Turin, Turin, Italy; 2 Laboratory of Cellular Biochemistry, Department of Animal Resource Sciences, Graduate School of Agriculture and Life Sciences, The University of Tokyo, Tokyo, Japan; 3 Department of Environmental, Biological and Pharmaceutical Sciences and Technologies, University of Campania “Luigi Vanvitelli”, Caserta, Italy

**Keywords:** circular RNA, miRNA, non-coding RNA, RNA sensing, RNA therapeutics, RNA world hypothesis, RNA-binding protein, transcriptome and epitranscriptome

RNA research is experiencing an unprecedented period of growth. Once regarded primarily as the intermediary between DNA and proteins, RNA is now recognized as a dynamic regulatory platform whose functions span virtually every level of biological organization, from a versatile regulator of gene expression to a key player in cellular identity, immunity, and disease. At the same time, advances in RNA engineering have established RNA molecules as powerful therapeutic agents, opening unprecedented opportunities for precision medicine. The articles collected in this Research Topic illustrate this exciting evolution of the field. They address diverse biological systems and diseases, spanning the continuum from fundamental concepts of RNA biology to its role and mechanistic studies in disease, as well as the emerging landscape of RNA-based therapeutics. Together they highlighting the extraordinary versatility of RNA and the challenges for its clinical translation.

Conceptually, the collection opens by placing RNA within the broadest possible biological framework. In their review, Flores-García et al. revisit the RNA World hypothesis by proposing the concept of multiple “RNA worlds” that connect three complementary regimes: the “primordial RNA world”, where RNA may have enabled the emergence of life through both information storage and catalysis; the “contemporary RNA world,” in which RNA regulates gene expression and cellular functions; and the “applied RNA world,” where RNA is engineered for biotechnology and medicine. The authors argue that these seemingly distinct fields share a common evolutionary principle: the relationship between RNA sequence, structure, and function under different environmental constraints. Understanding RNA through this unified framework not only advances origin-of-life research but also provides valuable design principles for RNA-based therapeutics, diagnostics, and synthetic biology.

A fundamental aspect of RNA function is its intimate relationship with the immune system. Henschel et al. review how cells distinguish self from non-self RNA to mount effective immune (e.g., antiviral) response while avoiding harmful autoimmunity. Their discussion highlights the importance of RNA chemical modifications and subcellular compartmentalization in preventing aberrant immune activation. Beyond providing insight into innate immunity, this work also illustrates why understanding RNA sensing has become essential for advancing next-generation RNA-based therapeutics, including mRNA vaccines.

The regulatory complexity of RNA is further expanded by the rapidly growing field of epitranscriptomics. Pernon et al. discuss the nuclear m6A reader YTHDC1 and its various roles in chromatin-associated regulation, transcriptional control, RNA processing, and nuclear condensate formation, with particular focus on hematological malignancies. By highlighting how alterations in RNA modifications and their recognition can markedly reshape gene regulatory networks, the authors demonstrate that epitranscriptomic regulation is at the center of both normal cellular homeostasis and cancer biology.

RNA-mediated regulation involves not only chemical modifications but, above all, the actions of distinct classes of RNA. Zainal Abidin et al. review the emerging biology of circular RNAs (circRNAs) in Down syndrome, an area that is significantly less studied than their involvement in cancer. They discuss how dysregulated circRNAs might affect processes like neuronal differentiation, synaptic function, chromatin remodeling, immune responses, and competing endogenous RNA networks (ceRNET). In particular, the review also highlights that much remains to be elucidated and that functional validation will be essential to determine impact of circRNA dysregulation on Down syndrome.

Original research papers go on to provide examples of the function of small non-coding RNAs in the regulation of disease-specific regulatory circuits. Wang et al. identifies that the RUNX1/miR-24/ALK4 axis plays an important role to activate hepatic stellate cells during liver fibrosis. The authors show that suppression of miR-24 induces profibrotic signaling mediated by ALK4 and Smad3 and propose that circulating miR-24 may be considered as both a biomarker and a potential therapeutic target. Similarly, Hu et al. investigate miR-18a-5p in the context of asthma-associated airway remodeling and demonstrate that this microRNA promotes airway smooth muscle cell proliferation and phenotypic changes through regulation of SPRY1 and activation of downstream signaling pathways, including the RAS-MAPK pathway. Despite focusing on different organs and diseases of interest, both studies demonstrate that microRNAs can act as crucial regulators linking extracellular signals with transcriptional and phenotypic responses. They also corroborate the idea that non-coding RNAs play an important role in complex pathological processes.

RNA-dependent disease mechanisms are not limited to regulatory RNAs themselves but also involve proteins that interact with RNA. The report by Geraci et al. examines RNA-protein interaction in Huntington’s disease by demonstrating neuron-specific overexpression of the RNA-binding protein MID1 in affected brains. Their work strengthens the hypothesis that aberrant interactions between MID1 and mutant huntingtin mRNA may contribute to disease progression. This further supports therapeutic strategies aimed at disrupting this pathogenic RNA-protein complex.

The power of transcriptomics as a discovery tool is illustrated by Zhao et al., who investigate the anti-inflammatory effects of Chinese herbal medicine *Polygonatum sibiricum* in an experimental loach model. RNA sequencing reveals widespread transcriptional remodeling following treatment and identifies Fcγ receptor-mediated phagocytosis as a potential mechanism underlying the observed anti-inflammatory activity. This study highlights the broad applicability of transcriptomic approaches for uncovering conserved molecular pathways and expanding RNA research beyond traditional biomedical models.

The collection concludes with a comprehensive overview of RNA-based therapeutics by Makkar. This review summarizes the progress achieved across multiple RNA platforms, including mRNA therapeutics, antisense oligonucleotides, small interfering RNAs, and RNA-editing technologies by CRISPR, while critically discussing the remaining challenges related to delivery, immune activation, manufacturing, and regulatory implementation. The author emphasizes that the future success of RNA medicine will depend not only on the development of new molecular platforms but also on overcoming practical barriers that limit widespread clinical applications.

Taken together, the articles in this Research Topic capture the breadth of contemporary RNA research, providing both a snapshot of current advances and a glimpse of future directions. They demonstrate that progress in the field increasingly depends on integrating fundamental discoveries in RNA biology with mechanistic insights into disease and innovative therapeutic strategies. From the origins of life to precision medicine, RNA research continues to redefine our understanding of biological regulation while providing powerful tools to address unmet clinical needs.

